# Effects of Green Network Management of Urban Street Trees on Airborne Particulate Matter (PM_2.5_) Concentration

**DOI:** 10.3390/ijerph20032507

**Published:** 2023-01-31

**Authors:** Na-Ra Jeong, Seung-Won Han, Baul Ko

**Affiliations:** Urban Agriculture Research Division, National Institute of Horticultural and Herbal Science, Wanju 55365, Republic of Korea

**Keywords:** urban green infrastructure, vegetation management, air quality, computational fluid dynamics (CFD), PM_2.5_ concentration, central reserve, street trees, wind characteristics

## Abstract

Street trees are crucial for air pollutant reduction in urban areas. Herein, we used computational fluid dynamics (CFD) simulation to identify changes in airborne particulate matter (PM_2.5_) concentration based on wind characteristics (direction and velocity) and the green network of street trees. The green network was assessed based on composition of the green area of street trees in the central reserve area and between the motor and pedestrian roads. The PM_2.5_ concentration varied according to the presence or absence of major reserve planting and the planting structure of the street trees, but not according to the wind direction or velocity. The concentration was lower when the wind direction was 45° (than when the wind direction was 0°), whereas it showed a more significant decrease as the wind velocity increased. Despite variation at each measurement site, the PM_2.5_ reduction was generally higher when the central reserve and street trees had a multi-planting structure. Hence, to ensure an effective reduction in the PM_2.5_ concentration on motor roads and reduce its negative impact on pedestrians, both arbors and shrubs should be planted in the central reserve area. The study results will serve as reference for managing the green area network and linear green infrastructure in terms of improving the atmospheric environment.

## 1. Introduction

In urban areas, air pollution is one of the major environmental factors that threaten human health [1]. One of the main causes of air pollution is the pollutants emitted by motor road traffic in urban areas [2]. Such pollutants may have a negative impact on the health of pedestrians using the road, people on bicycles, car drivers, and workers in buildings [3,4]. This prompted the development of various reduction policies for the improvement of air quality; notably, the strategy to reduce the discharge of pollutants was changed to a strategy to reduce the discharged pollutants. In recent times, as a passive measure, the use of plants has been one of the most widely applied methods to reduce pollutants [5,6]. Urban street trees contribute to the control of micro-climate in urban areas and the mitigation of urban heat islands [7,8,9,10,11,12], as the trees adsorb the pollutants [13,14,15,16,17,18] and exert a positive effect on the improvement of the urban environment. Despite these positive roles, the vegetation on motor roads could be an obstacle to the street canyon and may have an impact on airflow and pollutant dispersion, which may cause various problems [3,19,20,21].

The vegetation of urban street trees could affect the atmospheric air quality, due to its aerodynamic (as a porous obstacle that controls airflow) and pollutant reduction effects (via leaf surface deposition and porous absorption of pollutants, and the release of volatile compounds and pollens) [22,23,24,25,26]. The particulate pollutants are deposited on leaves, and the gas pollutants are absorbed by these leaves, leading to reduced pollutant concentration [13]. The aerodynamic effect of trees could be a complex phenomenon, depending on the environmental conditions, exerting a positive or negative effect on air quality. The airflow in the urban street tree environment may vary according to the street tree structure, climate conditions, and vegetation [5]. The factors influencing the structure of urban street trees include the heights and shapes of buildings and the width ratio of motor roads. The structural characteristics determine the airflow and the distribution of pollutants [27,28,29,30,31,32,33]. The pollutant concentration may also vary according to the wind direction and velocity [34,35], the vegetation location and type, the tree species, planting spacing, and canopy area [21,23,36,37,38,39,40].

The urban green infrastructure consists of different types of green spaces, including green points (that denote forests), green lines (that represent urban streets and green walls), and green planes of varying shapes, sizes, and structures in urban areas [41]. Each type consists of a green system based on the point-line-plane combination. In urban areas, the green areas of street trees are a vital part of the green infrastructure network, as they connect the green points and planes. A green area can be defined as a space containing plants on a natural or artificial ground in an outdoor space [42]. Green areas interact with airborne particulate matter via the individual plant elements or systems. Previous studies have analyzed the air improvement effect of green areas on varying scales, from an urban area or district scale [43] to a regional scale [44]. The presence of different dimensions of green areas has a complex effect on the distribution of air pollutants. In general, a green area modifies the trajectory, velocity, and other attributes of airborne particulate matter, along with their transient or permanent removal from the air [42].

To assess the effects of green areas of street trees on the quality of urban air, previous studies have used the methods of characterizing air current and pollutant dispersion from field measurements through numerical and wind tunnel simulations and outdoor reduction modeling [45]. Gromke et al. [46] conducted a wind tunnel simulation and computational fluid dynamics (CFD) analysis and concluded that when the overall concentration of traffic exhaust in street canyons with street trees was high, the flow velocity of wind decreased and that tree crown porosity had no significant effect on the pollutant concentration. Wania et al. [47] conducted a CFD analysis and reported that trees in street canyons reduced the wind velocity at the tree crown height and disturbed the flow fields near the canopy, which inhibited natural ventilation and increased the pollutant concentration. However, such negative effects of trees on the ventilation in street canyons should be treated as a trend unique to the investigated area because the street canyon composition and vegetation characteristics and contents vary in each area. Gromke and Ruck [21] indicated that an increase in the tree crown resulted in a decrease in the windward concentration, with a focus on the leeward concentration; they highlighted that the planting spacing was an important factor in the natural ventilation on motor roads. Huang et al. [40] indicated that the air pollutant flow and concentration in urban street canyons varied according to the street tree height. Li et al. [48] conducted field monitoring and numerical simulation to explain that vegetation barriers reduce the CO concentration on pedestrian and bicycle roads, with the suitable height of the barriers being 2.0 m. Buccolieri et al. [2] explained that the effect of trees varied according to the wind direction; a low width/height (W/H) ratio in a street canyon led to a significant increase in the effect of trees on the concentration increase in the vertical direction of the wind, and a high W/H ratio indicated a strong effect in the diagonal direction of the wind. In areas where the wind direction is parallel to the street canyon, the trees reduce the level of motor road traffic emission [49,50]. In a study that used CFD simulation for wind velocity, the ideal wind velocity for the pollutant deposition on the trees was 3 m/s [51]. Tong et al. [52] conducted a CFD analysis using a planting design, wherein the trees were planted before a robust barrier that was a combination of a wide vegetation barrier (having high leaf area density) and solid plant barrier; they proposed the model as a potential alternative solution for the mitigation of motor road pollutants. While some studies reported a negative effect of trees on the pollutant concentration in street canyons, others claimed that the effect of trees could vary depending on the wind direction, wind velocity, and street type. As several countries are currently promoting policies regarding street trees as an alternative measure to enhance the air quality of urban areas, a clear guideline should be developed for the construction and management of green areas to maximize the positive effect of vegetation on air quality.

Thus, the purpose of this study was to assess the potential effects of the green network of street trees on airborne particulate matter (PM_2.5_) concentration to provide basic data for setting an adequate guideline. We tested two variables in this study: (1) The changes in PM_2.5_ concentration were analyzed and quantified based on the planting composition; the urban green network of street trees was divided into the planting between the motor and pedestrian roads and the planting in the central reserve area. (2) The effects of the green network of street trees on PM_2.5_ concentration were analyzed, while considering the changes in the wind direction and velocity using CFD simulation. We aimed to verify two main hypotheses through these tests: (1) planting in the central reserve area will have a positive effect on the PM_2.5_ concentration; (2) the planting structure of street trees will affect the PM_2.5_ concentration.

## 2. Materials and Methods

### 2.1. Study Site

#### 2.1.1. Current Status

**Figure 1:**
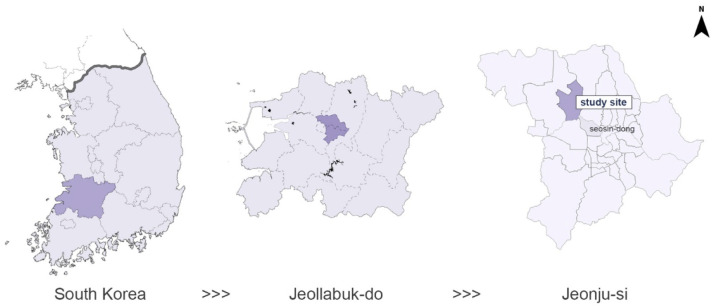
Map of the study area of Jeonju-si in Jeollabuk-do, South Korea.

In this study, we identified the planting structure of the green network of street trees in the Girin-daero region in Jeonju-si, Jeollabuk-do, South Korea; additionally, we studied the impact of the planting structure on the PM_2.5_ reduction in the region. A CFD simulation analysis was conducted, while considering the type of planting arrangement in the central reserve by controlling various influencing factors. The study site in Jeonju-si is located at 126°59′–127°14′ longitude and 35°43′–35°53′ latitude (Figure 1). Jeollabuk-do had the highest average concentration of PM_2.5_ in the country from 2016 to 2021. Jeonju is the central city of Jeollabuk-do, and during the same period, the average concentration of PM_2.5_ was the second highest in Jeollabuk-do.

#### 2.1.2. Climate Data

**Figure 2:**
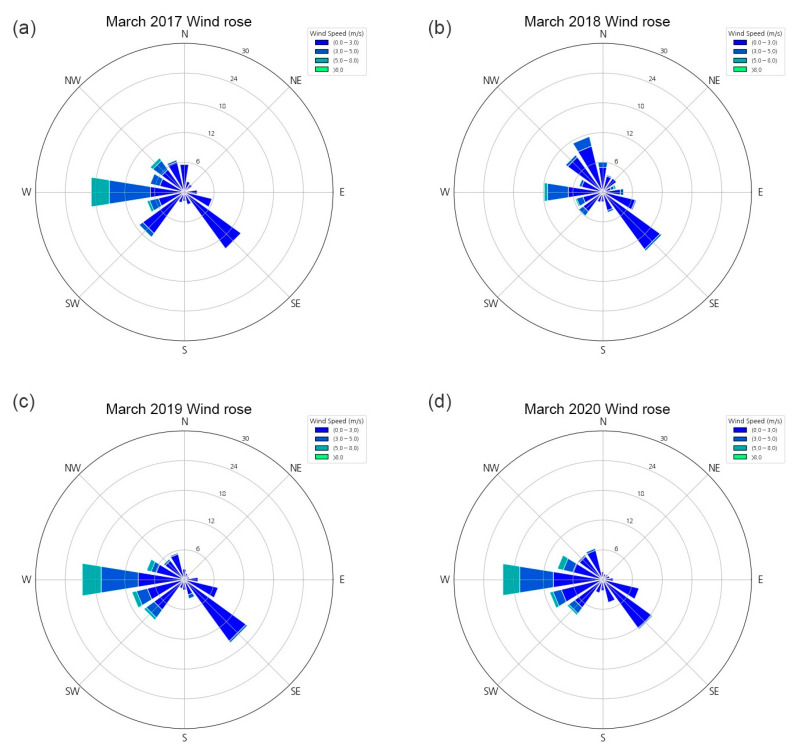
Number of days per wind class in March during 2017–2020: (**a**) 2017, (**b**) 2018, (**c**) 2019, and (**d**) 2020; the wind rose diagram was developed using Pycharm.

The climate data in this study were obtained from the Jeonju Office of Korea Meteorological Administration (KMA), which was located close to the study site. The data of the monthly Asian dust days and PM_2.5_ concentration for the study site revealed that March had the highest PM_2.5_ concentration and number of Asian dust days; therefore, we applied the climate data of March in the CFD simulation. To reflect the convection current due to the air and road temperatures, we considered the 10-year mean temperature for March (2011–2020), which was 7.2 °C. The mean and maximum wind velocities in the Jeonju-si region during 2017–2020 were 1.65 m/s and 7.2 m/s, respectively. In this study, we considered three scenarios of wind velocity in the CFD simulation: 1, 4, and 7 m/s. Based on the KMA data, the number of days per wind class (wind rose) was represented using Pycharm, a Python-integrated development environment (Figure 2), to set the wind direction as 0° and 45°.

For PM_2.5_ concentration, the data of PM_2.5_ concentration in March in recent years (2017–2019, 2021) were analyzed, and a mean of the highest 5% of concentrations was considered. The data of PM_2.5_ concentration were acquired from the Seosin-dong monitoring station, as its geographical characteristics were similar to those of the study site. The PM_2.5_ concentrations within the upper 5% were as follows: 82 μg·m^−3^ in 2017, 99 μg·m^−3^ in 2018, 148 μg·m^−3^ in 2019, and 75 μg·m^−3^ in 2021, with the mean concentration being 101 μg·m^−3^. The data for 2020 was missing due to the pilot operation of the PM_2.5_ measuring device at the Seosin-dong monitoring station.

### 2.2. Numerical Model

#### 2.2.1. Simulation Model of Tree Planting

**Figure 3:**
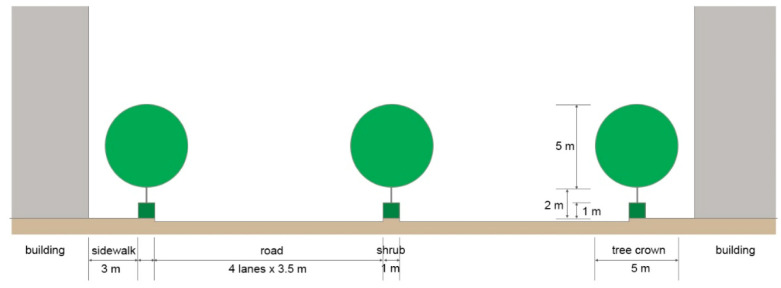
Illustration portraying the cross-section of the structure of the street and the specification of street trees.

**Figure 4:**
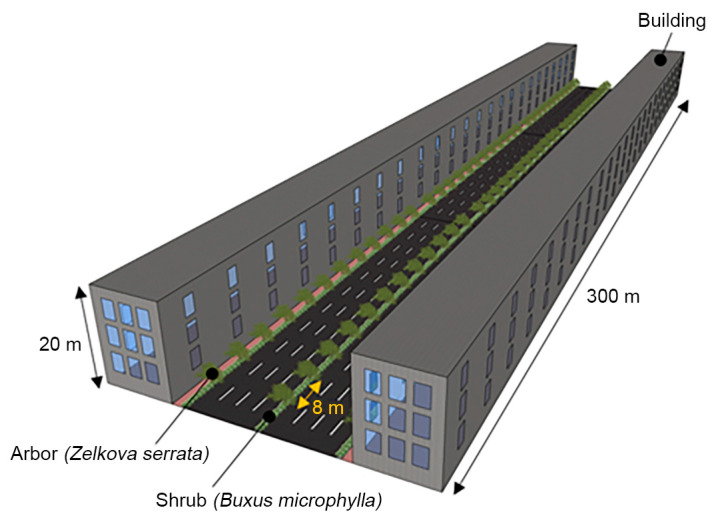
Simulation of the structure of the street and street trees.

In this study, we conducted a comparative analysis of six models based on the street tree and central reserve planting structures (Table 1). The cross-sectional structure of the motor road used for modeling consisted of a four-lane road, with buildings having heights of 20 m, a pedestrian road having a width of 3 m, and a green area of 1 m (with a 3.5 m width); the planting structure consisted of arbors (height 7 m) and shrubs (height 1 m). The spacing between the arbors was 8 m, according to the street tree planting criteria of the Street Tree Construction and Management Manual of the Korea Forest Service (Figure 3 and Figure 4).

The planting structure was set to a single-layer structure, wherein only arbors were planted, and a multi-layer structure, wherein shrubs were planted under the arbors to form layers. The arbor and shrub in the analysis were *Zelkova serrata* (Thunb.) Makino, which is a deciduous broad-leaved tree, and *Buxus microphylla* var. koreana, respectively. Of course, since the reduction effect of PM_2.5_ by trees varies depending on the vegetation cycle, coniferous trees show larger air pollution mitigation effects than deciduous trees [53]. However, in Korea, since the planting ratio of deciduous trees is high and various species of trees are used as street trees, experimental plants were selected among deciduous trees in this study. *Z. serrata* is suitable as a street tree in urban areas, as it has a strong wind resistance, rapid growth rate, strong resistance against pests and diseases, and beautiful fall foliage. A previous study has also shown that, compared to other street tree species, *Z. serrata* exhibits a strong effect of PM_2.5_ reduction [54]. Notably, *Z. serrata* is also a commonly found species as a street tree (27.1%) at the study site (Jeonju-si). In terms of the shrub analyzed for this study, *B. microphylla* is effective in PM_2.5_ reduction and has a high leaf area index (LAI) [55]. Like *Z. serrata*, *B. microphylla* can be planted anywhere in the country. It was also classified as an excellent grade in the PM reduction tree list released by the Korea Forest Service [56].

In the development of the criteria on central reserve and street tree planting, with the focus being its influence on PM_2.5_ reduction in urban outdoor spaces, first, it is important to analyze the windshield effect of planting; notably, the characteristics of the aerodynamic resistance caused by the planting should be accurately identified. The inertial resistance coefficient ($C_{2}$) can be calculated by using the drag coefficient ($C_{D}$), LAI, and tree height (*h*). The $C_{D}$ and LAI were based on the time when the tree growth was most vigorous. The LAI and $C_{D}$ of *Z. serrata* were reportedly 2.20 [54] and 0.61 [57], respectively, and those for *B. microphylla* were 4.54 [4,5,6,7,8,9,10,11,12,13,14,15,16,17,18,19,20,21,22,23,24,25,26,27,28,29,30,31,32,33,34,35,36,37,38,39,40,41,42,43,44,45,46,47,48,49,50,51,52,53,54,55] and 0.966, respectively [58]. We calculated the values of $C_{2}$ as 0.383 for *Z. serrata* and 8.771 for *B. microphylla* using the following formula.

$$C_{2}=2\times \frac{LAI}{h}\times C_{D}$$

These values were set as the $C_{2}$ of the porous media in the CFD simulation. In addition, to describe the PM_2.5_ reduction effect of the planting in the CFD analysis, the level of reduction of PM_2.5_ concentration was set to 50% on the Fluent for the PM_2.5_ passing through the porous media.

#### 2.2.2. Computational Fluid Dynamics (CFD) Model Design

In the CFD simulation used in this study, we applied the finite volume method to compute the concepts in the Reynolds theory of the Navier-Stokes equation for each cell on the internal and external motor road domains of the urban area. The main computational module was the Fluent (version 19.0., ANSYS Inc., Beltsville, MD, USA), and we used a 3-step analysis; pre-processing (to design the model exterior and form the mesh network regarding the target domain), main computation (to discretize the equation and obtain the solution using numerical analysis, by the computational domain of the designed model), and post-processing (to visually represent the simulation result). To develop a CFD model that could simulate the PM_2.5_ reduction due to planting, we applied the realizable k-ε turbulence model, which could predict the most adequate and proximate level of actual outcomes (in terms of spatial and temporal concentration profiles).

**Figure 5:**
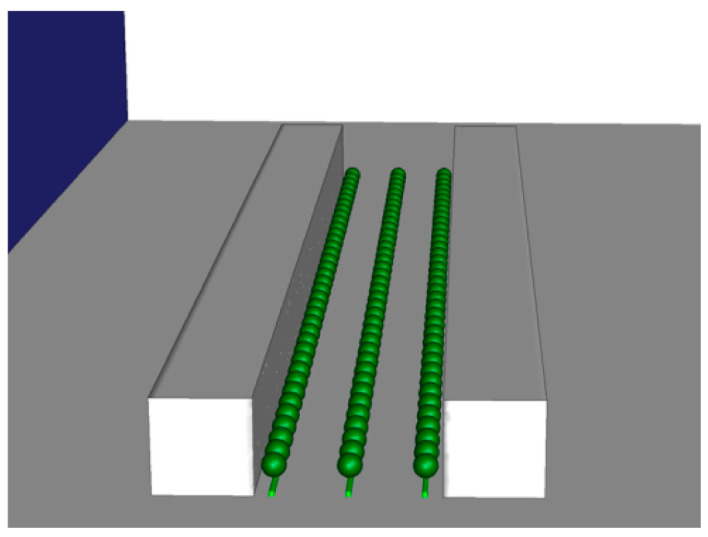
Illustration of the computational fluid dynamics (CFD) simulation model design applied in this study.

The geometry design was based on the data that met the simulation criteria, with the building width, height, and length being 20, 20, and 200 m, respectively, and the width and length of the motor road being 37 m and 140 m, respectively. For planting, the arbor crown width, height, and spacing were set as 5, 7, and 8 m, respectively, while the radius of the arbor crown (considered to be spherical) was set as 2.5 m. In the case of shrubs, a rectangular form was designed to line the streets and central reserve at a height of 1 m. The arbors were designed for 17 trees each, to make up 51 trees in total, and the shrubs were placed in a belt-form along the street and central reserve (Figure 5).

**Figure 6:**
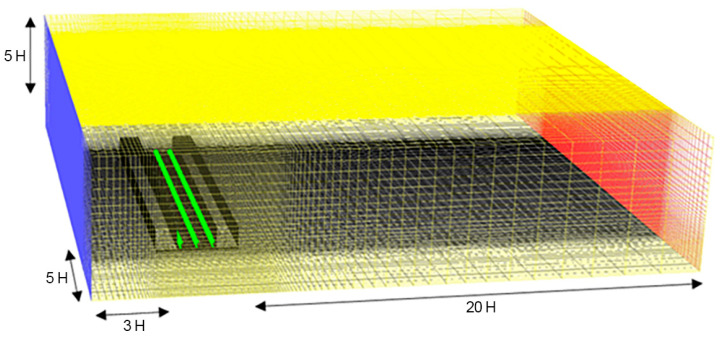
Mesh design of the computational fluid dynamics (CFD) simulation model used in this study (H indicates the height of a building).

The computational domain was designed in reference to two studies that conducted numerical predictions of the wind load on buildings, which contributed to the development of the wind load criteria by the Architectural Institute of Japan [59,60]. Note that the minimum leeward length should be ≥10 H, as at a length of ≤5 H, the reflux cannot be formed in the analytical domain and occurs at the boundary. Hence, the leeward length in this study was set as 20 H, to ensure that the wind pressure coefficient was constant, regardless of the leeward length. In addition, as the computational domain requires its sides to extend by ≥5 H on the external borders of the building, the lateral length, height, and wake length were set as 5 H, 5 H, and 3 H, respectively (Figure 6).

In the boundary conditions, the domain with the input of air current was set as the velocity inlet, and the domain with the output of air current was set as the pressure outlet. The floor of the analytical domain was set as the wall due to the presence of friction, while the top and the lateral sides were set to be symmetrical to increase the computation efficiency and scale a wide space to a finite space. The mesh size was 0.4 m for shrubs and 0.6 m for arbors, to enhance the accuracy and economic feasibility of the computation. For the domains, the sweep method was used to allow a gradual increase from 1.2 m to 1.5 m. The total number of meshes was 3,826,888 (Table 2).

For the CFD simulation, to create identical wind conditions, which would be similar to those in the Jeonju-si area, the wind profile of the average wind velocity, turbulence kinetic energy, and turbulence dissipation rate were applied in accordance with a previous study [61].

#### 2.2.3. Analytical Conditions

**Figure 7:**
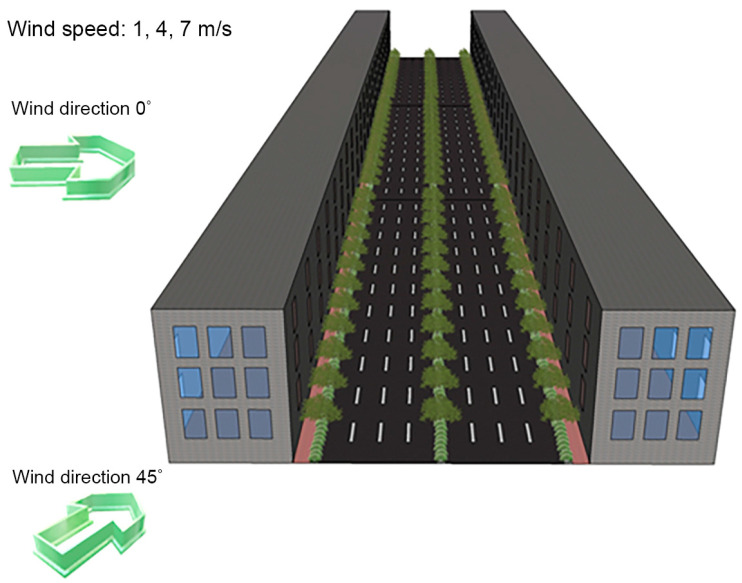
Illustration of the wind velocity and direction in the computational fluid dynamics (CFD) simulation model.

In this study, we analyzed the changes in the PM_2.5_ concentration in the study area for different models of urban street trees and central reserve planting based on the wind velocity and direction and planting structure. The analytical conditions were as follows: The wind velocity was considered as 1, 4, and 7 m/s, to reflect both the minimum and maximum wind velocities. We considered two wind flow directions, vertical (wind direction 0°) and diagonal (wind direction 45°) (Figure 7). Considering the PM_2.5_ matter floating in the air, the airflow, and the airborne dust rising from the motor road, we selected two planting types, namely arbor and shrub, to optimize PM_2.5_ reduction. To identify the most economic and efficient conditions of the planting structure, we analyzed the effect of the planting structure across the central reserve and the motor and pedestrian roads.

### 2.3. Analysis Methods

**Figure 8:**
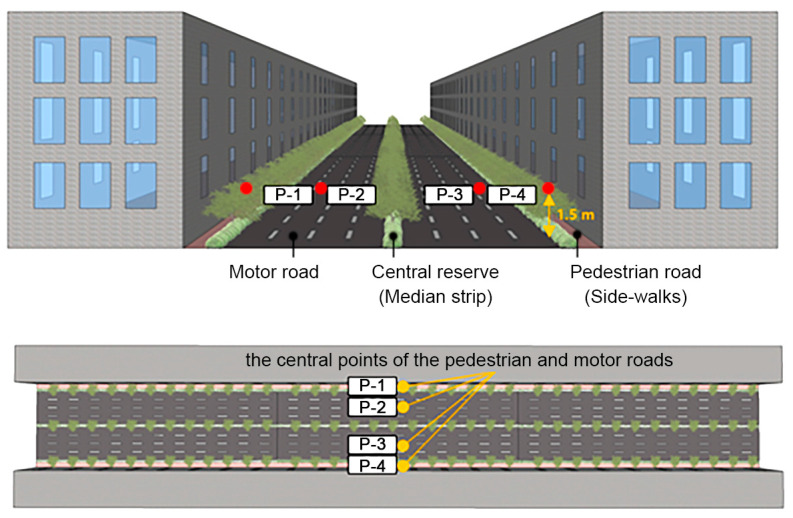
Measurement sites of particulate matter (PM_2.5_) concentration for the quantitative analysis conducted in this study; pedestrian road (P-1), leeward motor road (P-2), windward motor road (P-3), and windward pedestrian road (P-4).

To perform a quantitative analysis on the PM_2.5_ concentration in the study, the concentration data were collected from a total of four sites at the height of the breathing line (1.5 m) on the central cross-section for the wind direction of 0°. The four sites were the leeward pedestrian road (P-1), leeward motor road (P-2), windward motor road (P-3), and windward pedestrian road (P-4) (Figure 8).

## 3. Results

### 3.1. Changes in Particulate Matter (PM_2.5_) Concentration According to Wind Velocity

**Figure 9:**
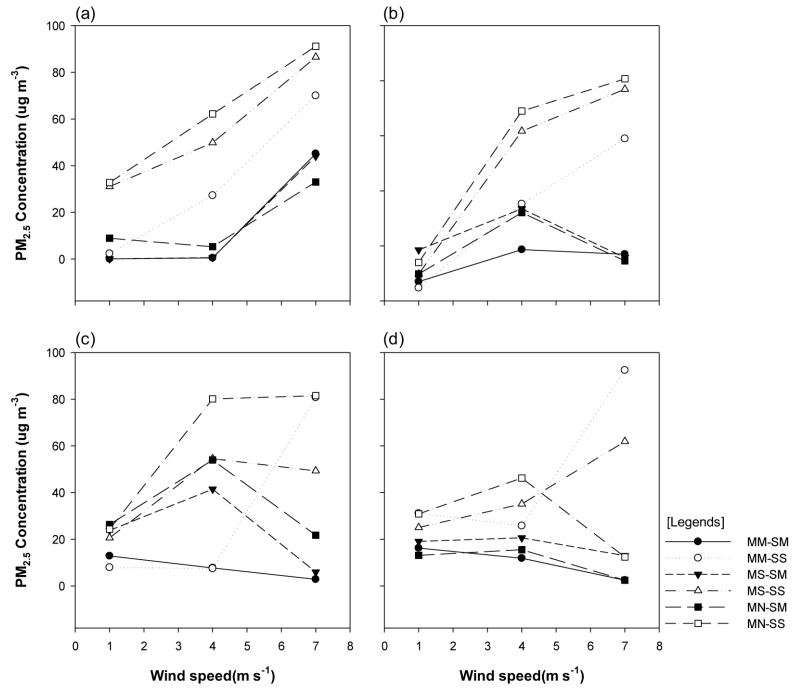
Graphs portraying the particulate matter (PM_2.5_) concentration at each site, based on the changes in the wind velocity, when the wind direction was vertical (0°): (**a**) pedestrian road (P-1), (**b**) leeward motor road (P-2), (**c**) windward motor road (P-3), and (**d**) windward pedestrian road (P-4).

We recorded the PM_2.5_ concentrations at four sites at the breathing line height of 1.5 m. The results indicated that, at P-1, the PM_2.5_ concentration portrayed an increasing trend with the increase in the wind velocity in the wind direction of 0°, regardless of the planting type. Notably, for the single-planting structure (SS), the PM_2.5_ concentration was ≤10 μg·m^−3^ for the wind speed scenarios of 1 m/s and 4 m/s, and ≥30 μg·m^−3^ for the wind speed of 7 m/s, which indicated that the concentration varied according to the wind velocity. At P-2, the PM_2.5_ concentration increased for the wind speeds of 1 m/s and 4 m/s in the MS-SM and MN-SM models, with a significant concentration reduction observed for the wind speed of 7 m/s. At P-3, the PM_2.5_ concentration rapidly decreased in the MM-SS model with increasing wind velocity, while the multi-planting structure of street trees (SM types) portrayed the highest PM_2.5_ concentration for the wind speed of 4 m/s. At P-4, the PM_2.5_ concentration of the planting types on the central reserve decreased with increasing wind velocity, regardless of whether the planting structure was SM or SS. The trend of PM_2.5_ concentration for the wind speed of 7 m/s was opposite to that for the wind speeds of 1 m/s and 4 m/s (where the PM_2.5_ concentration was markedly low in the MS-SM, MM-SM, and MN-SM models at P-1). For the wind velocity of 7 m/s, the PM_2.5_ concentration portrayed a notable reduction at P-4 (Figure 9).

**Figure 10:**
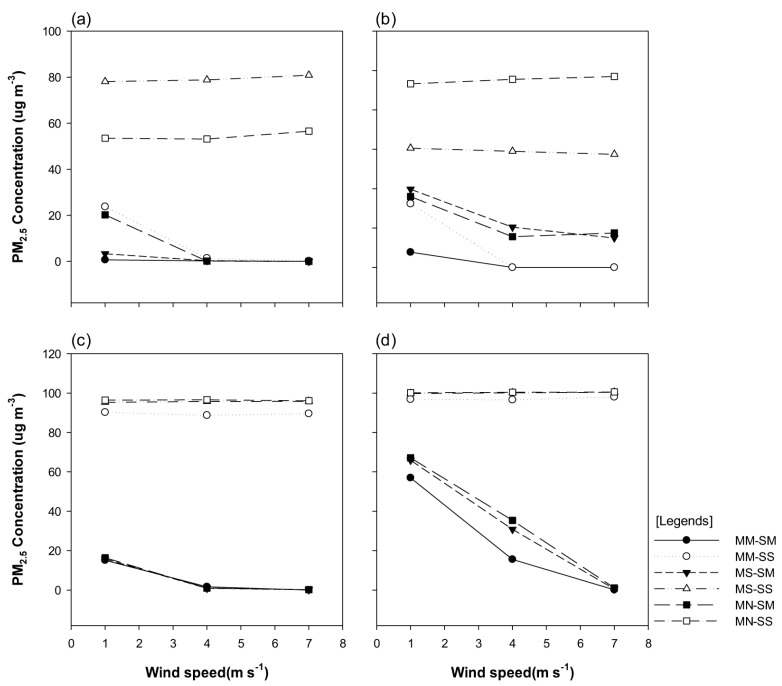
Graphs portraying the particulate matter (PM_2.5_) concentration at each site, based on the changes in the wind velocity for diagonal wind flow direction (45): (**a**) pedestrian road (P-1), (**b**) leeward motor road (P-2), (**c**) windward motor road (P-3), and (**d**) windward pedestrian road (P-4).

For the diagonal wind direction (45°), the PM_2.5_ concentration in the MN-SS and MS-SS models (with the street tree single-planting structure and either no or single-planting central reserve) was higher than that in other models (93.25–97 μg·m^−3^ and 57.46–60.56 μg·m^−3^ at P-1 and P-2, respectively, despite the increase in the wind velocity). At P-3 and P-4, at the right-hand side of the central reserve (in the windward domain), the PM_2.5_ concentration for the MS-SS, MM-SS, and MN-SS models, with the single-planting of street trees, stayed roughly constant (at 95–97 μg·m^−3^), despite the increase in the wind velocity. However, the PM_2.5_ concentration in the MS-SM, MM-SM, and MN-SM models (with the multi-planting of street trees) gradually decreased from 65.93 μg·m^−3^ to 1.0 μg·m^−3^ with increasing wind velocity. At P-4, we detected a large change in the PM_2.5_ concentration, corresponding to the wind velocity. As the wind velocity increased, the reduction in the PM_2.5_ concentration was high, although the change in the PM_2.5_ concentration was negligible at the wind velocities of 4 m/s and 7 m/s (Figure 10).

### 3.2. Changes in Particulate Matter (PM_2.5_) Concentration According to Wind Direction

Notably, the PM_2.5_ concentration was relatively high when the wind direction was considered as 45°. However, at P-1 and P-3, the PM_2.5_ concentration was below 90% for the wind direction of 45°.

**Figure 11:**
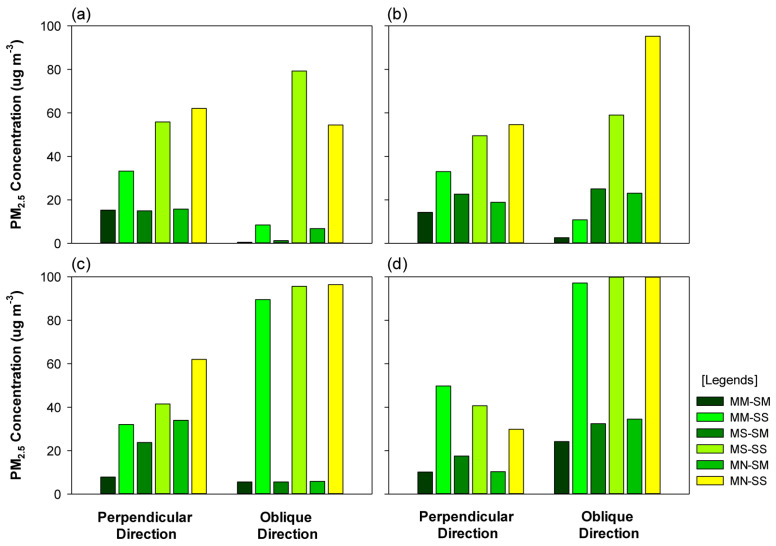
Graphs portraying the particulate matter (PM_2.5_) concentration at each site based on the changes in wind direction: (**a**) pedestrian road (P-1), (**b**) leeward motor road (P-2), (**c**) windward motor road (P-3), and (**d**) windward pedestrian road (P-4).

The variation in concentration was not significant at P-2. In contrast, at P-4, the PM_2.5_ concentration was high when the wind direction was 45°, for the models that adopted a multi-planting structure. For the wind direction of 45°, similar to the case where the wind direction was 0°, the PM_2.5_ concentration was lower in the SM models compared to the SS models (Figure 11).

### 3.3. Changes in Particulate Matter (PM_2.5_) Concentration According to Vegetation Structure

**Figure 12:**
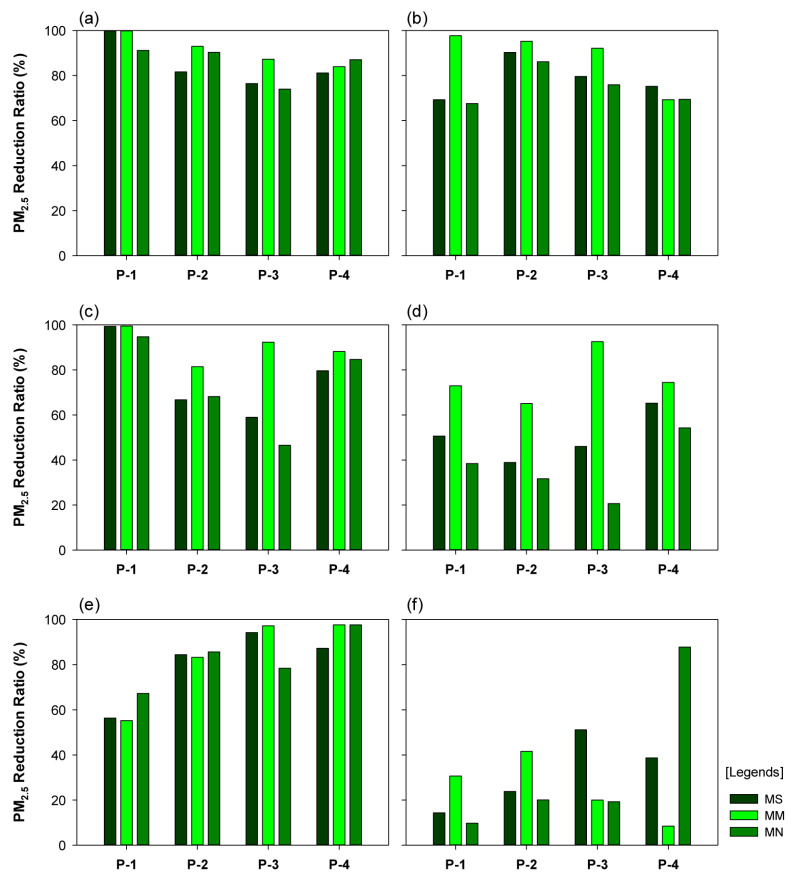
Graphs portraying the particulate matter (PM_2.5_) reduction rate, based on the street tree and central reserve planting structures, for the scenario where the wind direction was vertical (0°): (**a**) wind velocity of 1 m/s, with street tree-multi-planting (SM), (**b**) wind velocity of 1 m/s, with street tree-single planting (SS), (**c**) wind velocity of 4 m/s, with SM, (**d**) wind velocity of 4 m/s, with SS, (**e**) wind velocity of 7 m/s, with SM, and (**f**) wind velocity of 7 m/s, with SS; for the models, please refer to Table 1.

The changes in the PM_2.5_ concentration at the four sites (recorded at the breathing line height) were analyzed according to the central reserve and street tree planting models for the different conditions of wind direction and velocity (Figure 12).

For the wind direction of 0° and wind velocity of 1 m/s condition, the PM_2.5_ reduction rates were ≥90% at P-1 in the MS-SM, MM-SM, MN-SM, and MM-SS models; the reduction rates were 69.3% and 67.5% for the MS-SS and MN-SS models, respectively. The PM_2.5_ reduction rate at P-2 was high, with the reduction rates being 90.3% for the MN-SM model and 93.0% and 95.2% for the MM-SM and MM-SS models, respectively. In the MM-SS model, at P-3, the reduction rate was 92.1%, and relatively low rates were observed for the other models. At P-4, in all SM models, the reduction rates were relatively high, at 81.1%–87.0%. In the MM-SM model, the PM_2.5_ concentration was the lowest at all sites. In the MM-SS model, the reduction rate was high at all sites except P-4. In the MS-SM, MM-SM, and MN-SM models, the reduction rates were low at P-3 (close to the central reserve), which suggested a potential PM_2.5_ reduction effect of the multi-planting structure on the street trees and the central reserve.

In the -SM models, for the wind direction of 0° and wind velocity of 4 m/s, the PM_2.5_ reduction rates were 94.8–99.4% at P-1, indicating a markedly strong PM_2.5_ reduction effect. In the MM-SS model, the reduction rate was 73.0%. In the MM-SM, MS-SM, and MM-SS models, the PM_2.5_ reduction rates at P-2 were 81.5%, 66.8%, and 65.1%, respectively. In the MM-SS and MM-SM models, at P-3, the reduction rates were 92.6% and 92.4%, respectively, indicating a strong reduction effect of the central reserve planting. In the -SM models, at P-4, the reduction rates were 79.6–88.2%. Comparing the PM_2.5_ reduction rates in the MM-SM and MM-SS models showed a distinct difference.

For the wind direction of 0° and wind velocity of 7 m/s, the PM_2.5_ reduction rates were relatively high at all sites in the MN-SM, MS-SM, and MM-SM models, albeit with variations in the reduction rate at each site. Notably, the reduction rate was higher in the leeward domain than in the windward domain. At P-1 and P-2, the MN-SM model portrayed the highest reduction rates of 67.3% and 85.5%, respectively. At P-3 and P-4, the MM-SM model exhibited the highest reduction rates of 97.2% and 97.6%, respectively. The mean PM_2.5_ concentration across the four sites was 60.81 μg·m^−3^ for the MM-SS model and 20.12 μg·m^−3^ for the MM-SM model, indicating a large variation in the PM_2.5_ concentration in the two models. For P-4, the difference in the PM_2.5_ reduction rates was considerably high (at 79.4%) between the MN-SS and MN-SM models.

For the scenario with a wind direction of 45° and wind velocity of 1 m/s, the PM_2.5_ reduction rate at P-1 was the lowest in the MS-SS and MN-SS models (at 22.7% and 47.0%, respectively); the rate significantly decreased in other models as well. The PM_2.5_ reduction rate at P-2 in the MS-SS model decreased to 17.3%. In the MN-SS model, the reduction rate at P-3 was low at 53%. At P-3, compared to P-1 and P-2, we observed a distinct increase in the PM_2.5_ concentration. Finally, at P-4, we observed a similar trend to P-3, with a rapid fall in the PM_2.5_ reduction rate (to 34.7%, 43.6%, and 33.5% in the MS-SM, MM-SM, and MN-SM models, respectively). The results at the four sites indicated that, in the MM-SM model, which portrayed the strongest reduction effect, the PM_2.5_ reduction rate was 99.3% at P-1, with a considerably low numerical value of concentration, and 92.3%, 85.1%, and 43.6% at P-2, P-3, and P-4, respectively.

We compared the PM_2.5_ reduction rates of all six models based on the planting type; the reduction rates were low at multiple sites in the MS-SS model, with the lowest rate being 1.1% at P-4. For the MS-SM model, the PM_2.5_ concentration was relatively low at all sites compared to that observed in the MS-SS model. For the MM-SS model, the PM_2.5_ concentration decreased to 32.37 μg·m^−3^ at P-2, whereas the concentration increased to 23.80, 90.24, and 65.93 μg·m^−3^ at P-1, P-3, and P-4, respectively. Compared to that in the MM-SM model, the PM_2.5_ concentration was considerably high in the MN-SS model. The PM_2.5_ reduction rates in the MN-SM model at P-1, P-2, P-3, and P-4 were 80.0%, 64.4%, 83.8%, and 33.5%, respectively. Thus, the comparison between the MN-SM and MN-SS models indicated the effectiveness of the multi-planting structure in reducing PM_2.5_ concentration.

In the scenarios where the wind direction was 45°, and wind velocity was 4 m/s, the MM-SM model exhibited a noteworthy reduction in the PM_2.5_ concentration; the PM_2.5_ concentration was negligible at P-1 and P-2, 1.16 μg·m^−3^ at P-3, and 15.53 μg·m^−3^ at P-4. In the MS-SM, MM-SM, MN-SM, and MM-SS models, the PM_2.5_ reduction rate was significantly high at P-1. In the MS-SS and MN-SS models, the PM_2.5_ concentrations were 78.86 μg·m^−3^ and 53.15 μg·m^−3^, respectively; the variation in the PM_2.5_ concentration was higher at P-2 than at P-1. The PM_2.5_ reduction rate was low for models with no or single-planting central reserves. In the MS-SS and MN-SS models, the PM_2.5_ reduction rates at P-3 were low, at 5.1% and 4.8%, respectively. The PM_2.5_ concentrations were high for the models with no or single-planting central reserves. At P-4, compared to other sites, the PM_2.5_ reduction rate was relatively low. The PM_2.5_ reduction rate was higher in the MS-SS model, compared to that in the MN-SS model, but not significantly higher than that in the MS-SM and MM-SM models. Additionally, the PM_2.5_ reduction rate was higher in the MS-SM model, compared to that in the MN-SM model, but lower when compared to the reduction rate in the MM-SM model.

**Figure 13:**
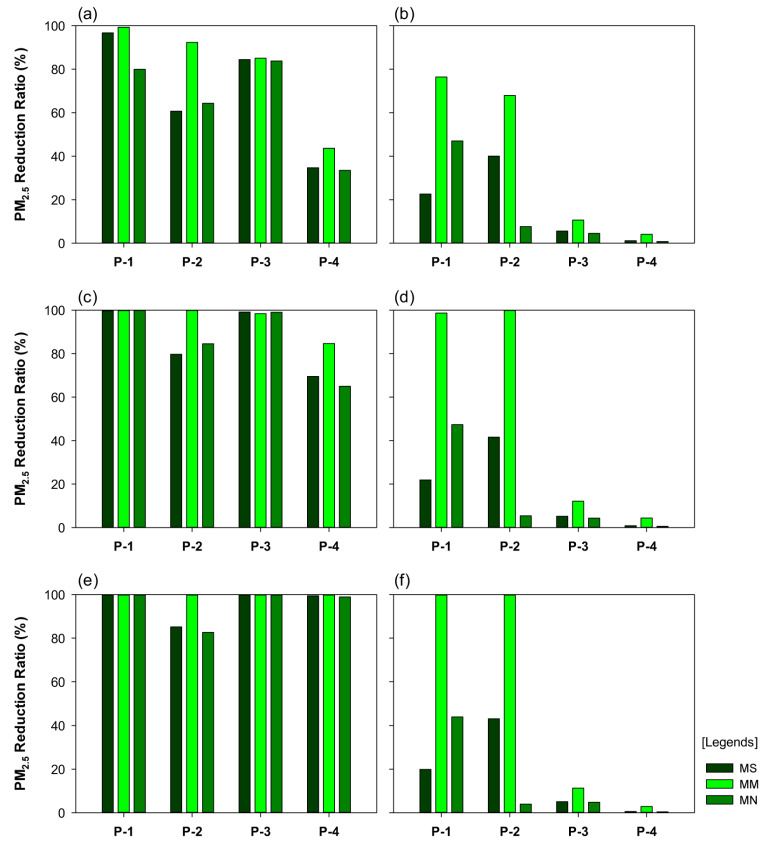
Graphs portraying the particulate matter (PM_2.5_) reduction rates for six scenarios and four sites, based on the street tree and central reserve planting structures, for the wind direction of 45°: (**a**) wind velocity of 1 m/s, street tree-multi-planting (SM), (**b**) wind velocity of 1 m/s, with street tree-single planting (SS), (**c**) wind velocity of 4 m/s, with SM, (**d**) wind velocity of 4 m/s, with SS, (**e**) wind velocity of 7 m/s, with SM, and (**f**) wind velocity of 7 m/s, with SS; for the types, please refer to Table 1.

In the scenario where the wind direction was 45°, and wind velocity was 7 m/s, in the MS-SS and MN-SS models, the PM_2.5_ reduction rates at P-1 were 19.9% and 44.0%, respectively; except for these two models, the PM_2.5_ reduction rates at P-1 were high in all other models. In the MS-SM and MM-SM models, the reduction rates were 100% for the sites, indicating low PM_2.5_ concentrations; the low PM_2.5_ concentrations were presumed to be due to the presence of the central reserve and the effect of the street tree multi-planting structure. In the MM-SS model, the average reduction rate was 99.8%; the low PM_2.5_ concentration could be attributed to the effect of the central reserve planting. In the MN-SM model, the average reduction rate was 100%; the low PM_2.5_ concentration could be attributed to the effect of the street tree multi-planting structure. The PM_2.5_ concentration was low, even though the model had no central reserve planting. In the MN-SM model, we observed a significant reduction in the PM_2.5_ concentration, despite the absence of the central reserve planting. At P-1, it is presumed that the street tree planting structure exerted a stronger effect on the PM_2.5_ reduction than the central reserve planting. At P-2, a trend similar to that in P-1 was observed in the MS-SM, MM-SM, MN-SM, and MM-SS models. In the MN-SM model, the PM_2.5_ concentration was relatively high at P-2. This may be due to the orientation of P-2 on the right side of P-1, when the wind directed at an angle of 45° caused an inflow of wind from P-4 to P-1. The graphs of P-3 and P-4 presented similar numerical values; the PM_2.5_ concentration was lower in the MS-SM, MM-SM, and MN-SM models, compared to that in the MS-SS, MM-SS, and MN-SS models (Figure 13).

## 4. Discussion

### 4.1. Changes in Particulate Matter (PM_2.5_) Concentration in Varying Climate Conditions

The width of the motor road and the direction of the wind had a significant effect on the pollutant concentration in the air. Buccolieri et al. [2] analyzed the impact of wind on the aerodynamic effects of trees regarding the pollutant concentration in street canyons and explained that a higher concentration was observed in street canyons when the wind direction was vertical; when the wind was in a diagonal direction, the pollutant reduction rate increased with the W/H ratio. In the vertical direction, two currents are created by the wind: a vortex at the center of the street and a vortex at the side/edge of the street. In such a case, the PM_2.5_ concentration is higher at the center of the street than at the side/edge of the street [8]. This is because, while the vortex at the center is the only possible region of air exchange at the center of the canyon, the overlap of the vortex at the center and the vortex at the edge can allow efficient ventilation at the edge of the street [21].

Furthermore, the aerodynamic effects of vegetation could decrease the wind velocity and increase the turbulence in the street [62]. The resistance of the vegetation can prevent airflow and pollutant dispersion; thus, the aerodynamic drag in the vertical direction is negative on the leeward wall, but positive on the windward wall [19]. In a street canyon, when the flow of the air is analyzed in accordance with the wind direction, a diagonal (oblique) input can induce more efficient ventilation [47]; however, an input in the vertical direction limits the ventilation in urban streets [63,64,65]. In this study, the scenario where the wind direction was 45° presented higher PM_2.5_ concentrations on the pedestrian road (lateral to the windward domain) compared to the scenario where the wind direction was 0°. For the models with the street tree single-planting structure and no central reserves, the large difference in concentration resulted in a relatively strong effect of the PM_2.5_ reduction rate on the pedestrian road. In the MM-SS and MM-SM models, the PM_2.5_ reduction effect could be detected when the central reserve had the multi-planting structure of arbor + shrub, regardless of the wind direction. Thus, a positive effect on the PM_2.5_ reduction rate could be predicted for the multi-planting structure of street trees in combination with the multi-planting structure in the central reserve.

Wania et al. [47] reported that a fall in the wind velocity suppressed and slowed down the vortices, and the air exchange and ventilation decreased as well. This consequently decreased the mixing of air within the street canyon and limited the input of fresh air. The effect of reduced wind velocity with a consequent increase in pollutants was analyzed by [20,46,66,67]. When the wind velocity ≤1.5 m/s, the vortices at the canyon disappeared, and the street air was stagnant [68]. Microparticles are dispersed through the air like gas particles, while large particles stay airborne for a short time [69]. Therefore, the vertical concentration gradient in the total suspended particles (TSP) is higher for larger particles than for microparticles [3]. In this study, we observed a decreasing trend in the PM_2.5_ concentrations on the pedestrian road with increasing wind velocity, regardless of the planting type. Vegetation served as an obstacle that reduced the airflow velocity, and a slow airflow caused a low air current and induced a low variation in the pollutant concentration (due to decreased dilution) [46,70]. In scenarios of poor ventilation, such as low wind velocity and vertical input, the variation in air quality is low due to the low variation in pollutant concentration [47]. For the models with no central reserves and single-planting structure, the variations in the PM_2.5_ concentrations were the highest, which may be due to the strong wind pushing away the PM_2.5_ particles and the relatively free wind flow underneath the street tree crowns.

In a low wind velocity condition below 1 m/s, the flow of pollutants cannot be sufficiently transferred to the trees. Under high wind velocity conditions, the flow of pollutants among the trees is rapidly carried out, causing less settlement [51]. In our study, in the models that adopted central reserve planting, an increase in wind velocity decreased the PM_2.5_ concentration. In the leeward domain, the PM_2.5_ reduction was affected to a greater degree by the street tree planting structure than by the central reserve planting structure. The negative vegetation effect of the leeward wall could be reduced using a planting structure composed of arbor + shrub, while the high-concentration pollution could be mitigated on both the leeward and windward walls [51]. Thus, the aerodynamic effect of vegetation on motor roads is critical in causing the changes in the flow field and, thus, in reducing the level of pollutants in street canyons.

### 4.2. Effects of Street Tree Planting in the Green Network on Particulate Matter (PM_2.5_) Concentration

In previous studies that carried out CFD simulation analysis, the wind in the vertical direction increased the pollutant concentration in the windward domain and decreased the concentration close to the leeward domain [3,71,72,73]. In this study, we observed a rapid change in pollutant concentration at P-4, according to wind velocity. In general, as the barrier formed on the lateral side of pedestrian roads in heavy traffic zones does not reconstruct the airflow within the street canyon, it can protect the pedestrian [74]. The barrier formed between a motor road and a pedestrian road prevents the dispersion of pollutants in traffic emissions [48,53,75,76]. On motor roads having a large width, the central reserve planting could create new vortices by forming a barrier that affects the airflow. Thus, to reduce the impact of PM_2.5_ on pedestrian roads, the central reserve planting is critical. In this study, at P-3 and P-4, in the windward domain on the right-hand side of the central reserve, the variations in the PM_2.5_ concentrations were not significant in the MS-SS, MM-SS, and MN-SS models, despite an increase in the wind velocity; in the MS-SM, MM-SM, and MN-SM models, the PM_2.5_ concentration portrayed a steady decrease with increasing wind velocity. The PM_2.5_ reduction rate was relatively high when the central reserve planting structure had a multi-planting model, regardless of the wind velocity or direction; the reduction rate was also high when the street tree planting structure had a multi-planting model.

Finally, compared to other models, the PM_2.5_ reduction effect was greater in the MS-SM, MM-SM, and MN-SM models for the wind direction of 45°; thus, to reduce the PM_2.5_ concentration on motor roads, the multi-planting structure consisting of both arbors and shrubs were deemed suitable as the street trees. The flow of air was affected by the planting of trees; thus, pollutants could accumulate on motor roads in high concentrations and come in contact with pedestrians [77]. However, at the breathing line height, shrubs could reduce the pollutant concentration through adsorption [78]. As shrubs could limit the dispersion of pollutants in the lower part of the street canyon, a greater amount of microparticles could pass through the vegetation [6]. Hence, short plants, such as shrubs, could be placed close to the emission source to filter out PM_2.5_, while tall arbors could increase the PM_2.5_ concentration by limiting their circulation and dilution in fresh air [13]. The arbor-shrub vegetation structure in the vicinity of highways exhibited the highest PM_2.5_ reduction rate [78]; as the shrubs in the tree-shrub planting composition limit the pollutant dispersion close to the ground surface, a large volume of pollutants could pass through the vegetation for accumulation, which will consequently reduce the PM_2.5_ concentration at the height of the pedestrian breathing line to 16.5–20.6% [9].

Compared to the single-planting structure of the street tree arrangement adopted in this study, the multi-planting structure portrayed a significantly stronger PM_2.5_ reduction effect; in the presence of the central reserve planting, the PM_2.5_ reduction rate was even higher. In the absence of the central reserve planting, the variation in the PM_2.5_ reduction rate between the single-planting and multi-planting structures was significantly high. This accounted for the PM_2.5_ reduction effect on the side of the pedestrian road, even in the absence of the central reserve planting. The multi-planting structure of shrubs and arbors between the motor and pedestrian roads served as a hedge that created a boundary between the roads. Such hedge structures have a potential positive role in improving the air quality for pedestrians. In field studies that employed barrier formations between pedestrian roads and motor roads, the pollutant concentration on the side of the pedestrian roads was 27–52% lower than that on the side of the motor roads [53,75]. In simulation studies, the reported pollution level was 26–41% lower on the side of the pedestrian road than on the side of the motor road [23,48,73]. A good strategy to minimize the impact of PM_2.5_ on pedestrians is to create a vertical plane, to prevent the input of PM_2.5_ from the motor roads to the pedestrian roads [73]. A planting design is also required that can provide a large planting surface close to the emission source without affecting the air exchange [6].

## 5. Conclusions

In this study, we conducted a CFD simulation to analyze the changes in the PM_2.5_ concentration of a street, based on the green network of street trees and wind characteristics. The green network of the street trees was assessed through the planting composition of the green area of the street trees between the motor and pedestrian roads, and on the central reserve. For the wind characteristics, the effects of wind direction and velocity were investigated. The findings of this study are as follows:

1. Analyzing the PM_2.5_ concentration irrespective of wind direction or velocity indicated that the PM_2.5_ concentration varied according to the presence or absence of the central reserve and the street tree planting structure. The PM_2.5_ concentration was lower in the wind direction of 45° than in the wind direction of 0°; the deviation of the PM_2.5_ concentration per planting type increased with the wind velocity.
2. Despite the numerical differences across the study sites, the PM_2.5_ reduction effect at most sites was stronger in the models that adopted multi-planting street trees and central reserves. These results proved the hypothesis (the planting structure of street trees and central reserve area will affect the PM_2.5_ concentration).
3. Finally, the application of the central reserve planting was more advantageous in reducing the PM_2.5_ concentration on motor roads; we suggest planting both arbors and shrubs as street trees. As the PM_2.5_ reduction effect was stronger when the central reserve contained arbors and shrubs, the multi-planting structure should be applied to both the street trees and the central reserve, to ensure an optimal PM_2.5_ reduction effect. Hence, the planting of both arbors and shrubs is more suitable for reducing the negative impact of PM_2.5_ on motor roads.

The findings of this study support the public healthcare effect of green areas while providing a practical guideline for the construction and management of urban green infrastructures. However, this study has a few limitations. The types of street trees applied in the simulation were limited, phenological characteristics were not considered, and the changes in the PM_2.5_ concentration were quantified without considering the mechanisms of the plants for reducing PM_2.5_ (e.g., dispersion and absorption). Further studies should conduct a more in-depth analysis of the PM_2.5_ reduction effect while considering the green network of street trees and the planting composition, focusing on specific mechanisms (e.g., pollutant dispersion and adsorption) to provide a more specified guideline on street tree planting in urban areas.

## Figures and Tables

**Table 1:** Details of the six models compared in this study.

Type	Central Reserve	Street Tree
MS-SS	Single-planting structure (arbor)	Single-planting structure (arbor)
MS-SM	Single-planting structure (arbor)	Multi-planting structure (arbor + shrub)
MM-SS	Multi-planting structure (arbor + shrub)	Single-planting structure (arbor)
MM-SM	Multi-planting structure (arbor + shrub)	Multi-planting structure (arbor + shrub)
MN-SS	N/A	Single-planting structure (arbor)
MN-SM	N/A	Multi-planting structure (arbor + shrub)

**Table 2:** Conditions considered in the computational fluid dynamics (CFD) simulation model design applied in this study.

Category	Setting of Model
Solver	Pressure based
	Implicit formulation
	Unsteady state analysis
	3D simulation
Turbulence model	Realizable *k-ε* model
Wind profile	Velocity inlet, User-Defined-Function (C code)
Planting	Porous media
Domain	Symmetry

Note: three dimensional (3D).

## Data Availability

Not applicable.
